# Treadmill Exercise-Induced RNA m6A Methylation Modification in the Prevention of High-Fat Diet-Induced MASLD in Mice

**DOI:** 10.3390/ijms26125810

**Published:** 2025-06-17

**Authors:** Xueli Liu, Yuanming Zhong, Yuqian Guo, Jianhua Xu, Shaobing Wang, Yiping Liu, Yi Lv, Xi Zheng

**Affiliations:** 1 Fujian Key Laboratory of Developmental and Neural Biology, College of Life Sciences, Fujian Normal University, Fuzhou 350117, China; m18159963049_1@163.com (X.L.); guoyuqian0628@163.com (Y.G.); 2 School of Physical Education and Sport Sciences, Fujian Normal University, Fuzhou 350117, China; 13007958372@163.com (Y.Z.); xujianhua@fjnu.edu.cn (J.X.); 3 Provincial University Key Laboratory of Sport and Health Science, School of Physical Education and Sport Sciences, Fujian Normal University, Fuzhou 350117, China; wshbingcn@fjnu.edu.cn (S.W.); ypliu1966@126.com (Y.L.)

**Keywords:** aerobic exercise, RNA-Seq, MeRIP-seq, lipid metabolism, *Paqr7*

## Abstract

Exercise is a well-recognized non-pharmacological strategy for preventing and managing metabolic dysfunction-associated steatotic liver disease (MASLD, formerly known as NAFLD). While the benefits of exercise are thought to involve epigenetic mechanisms, the precise role of RNA m6A methylation remains unclear. This study investigates how treadmill exercise modulates RNA m6A methylation to prevent MASLD in a high-fat diet (HFD)-induced mouse model. Male C57BL/6 mice were fed either a standard diet (SD) or HFD for 12 weeks, with a subset of HFD-fed mice undergoing treadmill exercise (HFD + Ex). Liver pathology and biochemical markers were assessed. RNA sequencing (RNA-Seq) and methylated RNA immunoprecipitation sequencing (MeRIP-seq) were performed to identify differentially expressed genes (DEGs) and m6A methylation changes. Key candidate gene *Paqr7* was validated through siRNA-mediated knockdown in AML-12 cells to assess its role in lipid metabolism. Treadmill exercise alleviated MASLD-related pathology and biochemical abnormalities. RNA-Seq identified 984 DEGs in the HFD vs. SD comparison and 544 in the HFD + Ex vs. HFD comparison. Intersection analysis identified 155 genes upregulated in MASLD and downregulated following exercise. MeRIP-seq revealed 225 hypermethylated and 208 hypomethylated m6A peaks in HFD + Ex vs. HFD groups. Integrative analysis highlighted *Adra2b*, *Lipa*, and *Paqr7* as key exercise-responsive genes. Silencing *Paqr7* through siRNA-mediated knockdown reduced lipid accumulation and suppressed lipogenic gene expression, suggesting its role in exercise-mediated MASLD improvement. Treadmill exercise prevents MASLD by modulating RNA m6A methylation, with *Paqr7* emerging as a potential regulator of lipid metabolism. These findings highlight epigenetic modulation as a key mechanism in exercise-induced liver protection.

## 1. Introduction

Physical inactivity is a major risk factor for the onset and progression of metabolic dysfunction-associated steatotic liver disease (MASLD, formerly known as NAFLD). MASLD encompasses a spectrum of conditions ranging from simple steatosis to liver inflammation, fibrosis, and end-stage cirrhosis [1]. The increasing prevalence of MASLD is closely associated with sedentary lifestyles, which have become more common due to shifts in work patterns and reduced physical activity in modern societies [2]. Coupled with the rising incidence of obesity and metabolic syndrome, which are key drivers of MASLD, this condition has become the most common cause of chronic liver disease globally, affecting an estimated 25% of the population [3]. Lifestyle interventions, particularly those incorporating physical activity, are fundamental to both the prevention and management of MASLD [4,5]. Aerobic exercise has been shown to enhance liver function by reducing hepatic fat deposition, improving insulin sensitivity, and lowering liver enzyme levels, even without significant weight loss [6,7,8]. However, the molecular mechanisms underlying these beneficial effects remain poorly understood. A growing body of evidence suggests that epigenetic modifications, which mediate the interaction between environmental factors such as exercise and gene expression, could play a pivotal role in mitigating MASLD [9].

Epigenetic changes refer to heritable modifications in gene expression that occur without alterations in the DNA sequence. These include DNA methylation, histone modifications, and regulation by non-coding RNAs [10]. Recently, N6-methyladenosine (m6A) methylation, the most abundant internal modification of messenger RNA (mRNA) in eukaryotic cells, has emerged as a crucial regulator of gene expression [11,12]. Importantly, m6A methylation has been implicated in regulating metabolic processes, including lipid metabolism, inflammation, and oxidative stress, which are central to the pathogenesis of MASLD [13,14,15]. While research has increasingly focused on the role of m6A methylation in metabolic diseases, the specific impact of exercise on this epigenetic mechanism in MASLD remains poorly characterized. Data from both clinical and animal studies indicate that exercise influences physiological processes through m6A modifications across various systems, including skeletal muscle, the nervous system, the circulatory system, and the reproductive system [16,17,18,19,20]. However, research exploring its effects on hepatic function remains scarce.

Given the established benefits of exercise on MASLD and the emerging understanding of m6A methylation in metabolic regulation, it is conceivable that exercise-induced modulation of RNA m6A methylation may serve as a key mechanism through which physical activity exerts its protective effects against MASLD. This study aims to explore how treadmill exercise alters hepatic m6A methylation in a mouse model of HFD-induced MASLD. We hypothesized that exercise may influence hepatic m6A methylation patterns and thereby affect the expression of genes involved in lipid metabolism, inflammation, and oxidative stress—three key processes in the development and progression of MASLD. Elucidating these key genes and molecular pathways may uncover new therapeutic targets for MASLD and reinforce the role of exercise as a non-pharmacological strategy in its prevention and treatment.

## 2. Results

### 2.1. Treadmill Exercise Mitigates HFD-Induced Weight Gain and Liver Dysfunction in Mice

**Figure 1:**
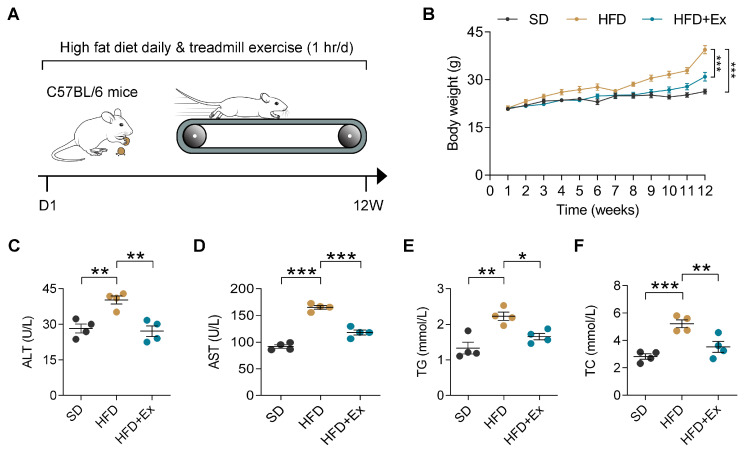
Treadmill exercise prevents body weight gain, liver damage, and dyslipidemia in HFD-fed mice. (**A**) Schematic of the experimental design: C57BL/6J mice were subjected to a standard diet (SD) or high-fat diet (HFD) for 12 weeks, with or without daily treadmill exercise (1 h/day). D1: Day 1; 12w: 12 weeks. (**B**) Weekly body weight measurements showed significant weight gain in HFD-fed mice compared to the SD group. Treadmill exercise significantly reduced body weight gain in the HFD + Ex group compared to the sedentary HFD group (*n* = 6 mice per group). (**C**–**F**) Serum biochemical analysis revealed increased levels of alanine aminotransferase (ALT), aspartate aminotransferase (AST), triglycerides (TG), and total cholesterol (TC) in the HFD group compared to the SD group. Exercise significantly reduced these markers in the HFD + Ex group compared to sedentary HFD mice (*n* = 4 mice per group). Data are presented as mean ± SD. * *p* < 0.05, ** *p* < 0.01, *** *p* < 0.001 by one-way ANOVA.

To evaluate the protective effects of exercise against MASLD, C57BL/6 mice fed with HFD were subjected to an established protocol of aerobic exercise (treadmill training) for 12 weeks (Figure 1A). During the 12-week intervention, the body weight of HFD-fed mice was markedly higher compared to those on a standard diet, while the HFD + Ex group exhibited significantly reduced weight gain relative to the sedentary HFD group (Figure 1B). In addition, serum levels of liver enzymes alanine aminotransferase (ALT) and aspartate aminotransferase (AST) were elevated in HFD-fed mice, indicating liver injury, but these levels were significantly lower in the HFD + Ex group (Figure 1C,D). Likewise, triglycerides (TG) and total cholesterol (TC) levels were significantly increased in HFD-fed mice, with treadmill exercise reducing the level (Figure 1E,F). Overall, these data underscore the efficacy of treadmill exercise in alleviating the systemic and hepatic metabolic consequences of HFD.

### 2.2. Treadmill Exercise Suppresses MASLD Development in HFD-Fed Mice

**Figure 2:**
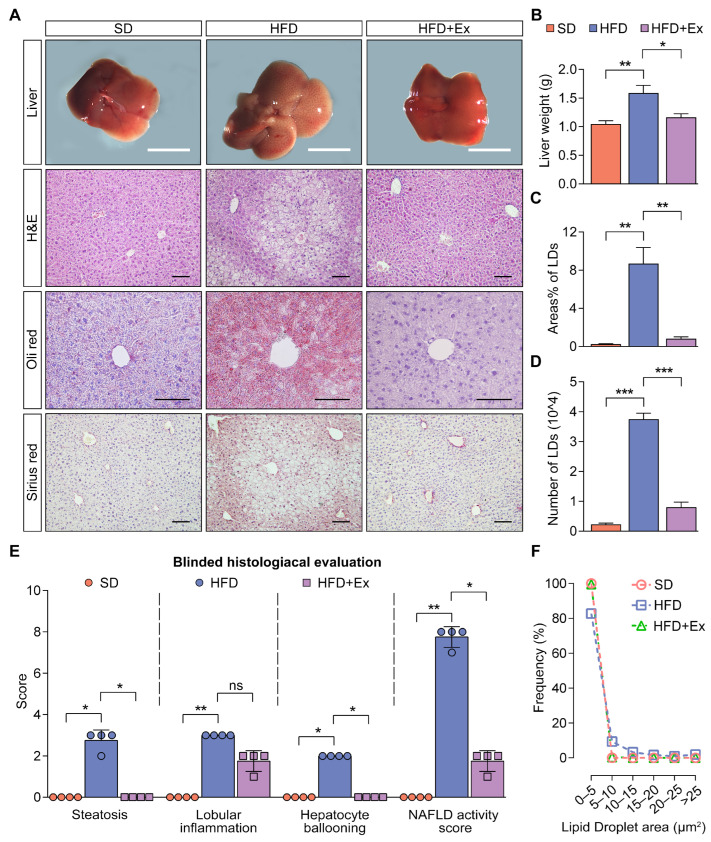
Treadmill exercise alleviates liver pathology and lipid accumulation in HFD-induced MASLD mice. (**A**) Representative images of gross liver morphology, H&E, Oil Red O, and Sirius Red-stained liver sections from SD, HFD, and HFD + Ex groups. Scale bars = 1 cm for gross liver images and 100 µm for histological sections. (**B**) Liver wet weight is significantly increased in HFD-fed mice compared to the SD group, with a reduction in the HFD + Ex group (*n* = 6 mice per group). (**C**) Quantification of lipid droplet (LD) area as a percentage of total liver area, and (**D**) the number of LDs per field of view in liver sections stained with Oil Red O (*n* = 3 mice per group). (**E**) Blinded histological evaluation of steatosis, lobular inflammation, hepatocyte ballooning, and NAFLD activity score (*n* = 4 mice per group). (**F**) Frequency distribution of lipid droplet size (µm2) in liver sections. Data are presented as mean ± SD. * *p* < 0.05, ** *p* < 0.01, *** *p* < 0.001 by one-way ANOVA (**B**–**D**) or by Kruskal-Wallis test (**E**).

Gross examination of liver morphology showed that HFD-fed mice exhibited visibly larger, discolored livers compared to the SD group, whereas the HFD + Ex group had visibly healthier livers (Figure 2A, top row). Liver wet weight was significantly increased in HFD-fed mice compared to the SD group, and this increase was reduced by treadmill exercise (Figure 2B). Histological analysis confirmed that HFD-fed mice had severe hepatic lipid droplet accumulation, inflammation, and fibrosis, which were ameliorated by exercise, as evidenced by H&E, Oil Red O, and Sirius Red staining (Figure 2A,C,D). Additionally, blinded histological evaluation indicated significantly higher NAS in HFD-fed mice, with notable improvements in steatosis and hepatocyte ballooning in the HFD + Ex group, while lobular inflammation showed a non-significant trend toward reduction (Figure 2E). Lipid droplet size distribution analysis revealed that the HFD group exhibited a higher proportion of large lipid droplets, which were significantly reduced by treadmill exercise (Figure 2F). Collectively, these findings demonstrate that treadmill exercise effectively mitigates the hallmark features of MASLD.

### 2.3. Treadmill Exercise Modulates Hepatic Gene Expression and Alters Metabolic Pathways in HFD-Induced MASLD

**Figure 3:**
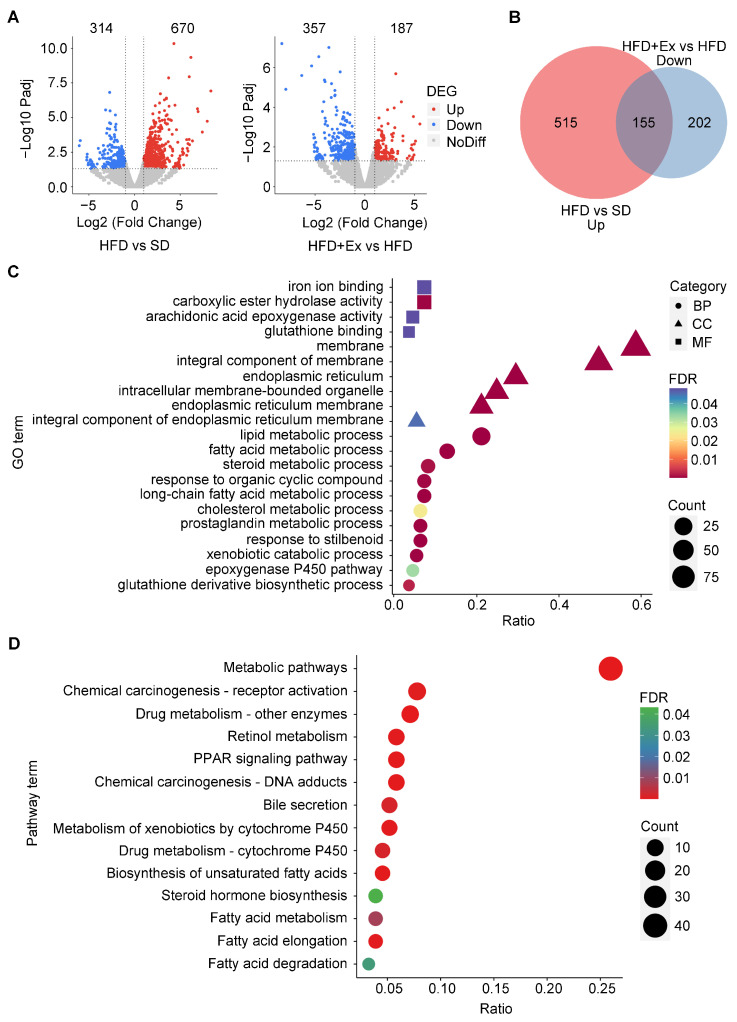
Transcriptomic changes in the liver induced by HFD and treadmill exercise. (**A**) Volcano plots showing differentially expressed genes (DEGs) in the liver of HFD vs. SD (left) and HFD+Ex vs. HFD (right) mice. Upregulated (red) and downregulated (blue) genes are shown. (**B**) Venn diagram indicating overlapping genes between those upregulated in HFD vs. SD and downregulated in HFD+Ex vs. HFD (*n* = 155 genes). (**C**) Gene ontology (GO) analysis of the 155 overlapping genes, categorized into biological processes (BPs), cellular components (CCs), and molecular functions (MFs). Dot size represents gene count, and color indicates false discovery rate (FDR). (**D**) KEGG pathway enrichment analysis of the same 155 genes. Data are presented with FDR-adjusted *p*-values and gene ratios.

To understand the molecular mechanisms underlying the protective effects of treadmill exercise on liver function, we performed RNA sequencing of liver samples from the SD, HFD, and HFD + Ex groups. Transcriptomic analysis revealed significant dysregulation of gene expression in the livers of HFD-fed mice, with 670 upregulated and 314 downregulated genes compared to the SD group. In the HFD + Ex group, treadmill exercise markedly altered the gene expression profile compared to sedentary HFD mice, with 187 genes significantly upregulated and 357 downregulated (Figure 3A). Among these, 155 genes were specifically downregulated by treadmill exercise after being upregulated by HFD (Figure 3B). Gene ontology (GO) enrichment analysis of these 155 genes revealed significant associations with biological processes (e.g., fatty acid metabolic process, steroid metabolism), cellular components (e.g., endoplasmic reticulum membrane), and molecular functions (e.g., glutathione binding and iron ion binding), indicating a restoration of lipid metabolic homeostasis and endoplasmic reticulum integrity by treadmill exercise (Figure 3C). Similarly, KEGG pathway enrichment analysis highlighted the reversal of dysfunctions in key pathways, including fatty acid metabolism, the PPAR signaling pathway, bile secretion, and drug metabolism, all of which are critical for maintaining liver function and mitigating lipid accumulation (Figure 3D). These results demonstrate that treadmill exercise counteracts HFD-induced hepatic transcriptomic dysregulation by restoring specific molecular pathways involved in lipid metabolism and detoxification processes, including glutathione binding, iron ion binding, drug metabolism, and bile secretion.

### 2.4. Treadmill Exercise Modulates Hepatic m6A RNA Methylation in HFD-Induced MASLD

**Figure 4:**
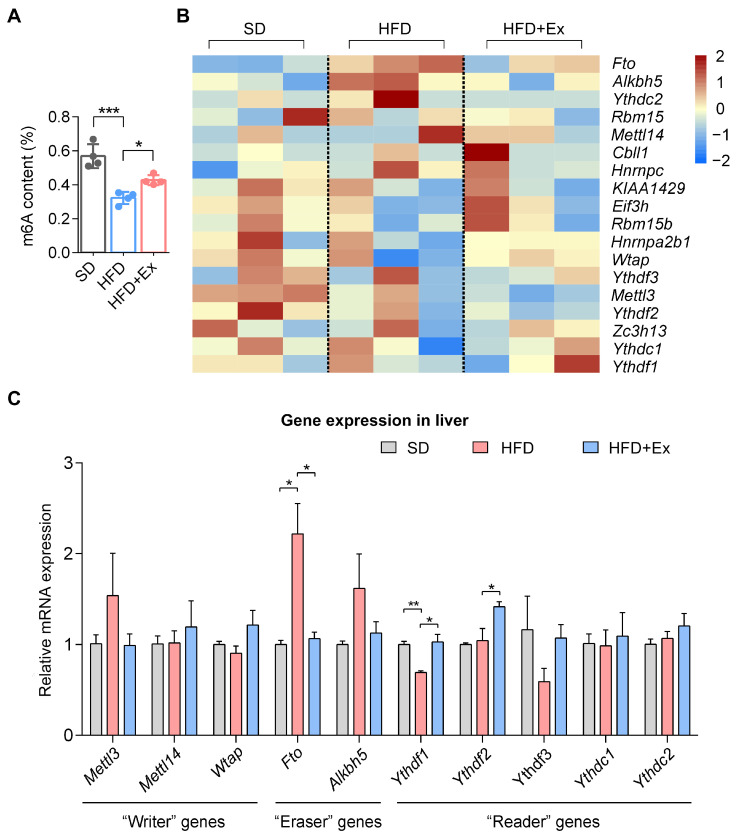
Treadmill exercise modulates hepatic m6A methylation and alters expression of m6A-associated genes in HFD-fed mice. (**A**) Global m6A content (% of total RNA) in the liver across SD, HFD, and HFD + Ex groups (*n* = 3 mice per group). (**B**) Heatmap showing the expression profiles of key m6A “writer”, “eraser”, and “reader” genes in the liver. Expression values are normalized and scaled (*n* = 3 mice per group). (**C**) Relative mRNA expression of m6A regulatory genes quantified by RT-PCR in liver tissues (*n* = 3 mice per group). * *p* < 0.05, ** *p* < 0.01, *** *p* < 0.001 by one-way ANOVA.

To explore whether the change in gene expression is related to m6A RNA methylation, we examined the effects of treadmill exercise on m6A RNA methylation levels and the expression of m6A-modifying genes in the livers of mice subjected to an HFD. Global m6A methylation levels were significantly reduced in the HFD group compared to the SD group. However, treadmill exercise reversed this effect, restoring m6A levels close to those observed in the SD group (Figure 4A). Gene expression analysis of m6A “writers”, “erasers”, and “readers” revealed distinct patterns among treatment groups. The heatmap shows that HFD altered the expression of multiple m6A regulatory genes, with key changes in *Fto* and *Alkbh5* (erasers) and *Ythdf1* and *Ythdf2* (readers) (Figure 4B). Exercise intervention normalized several of these changes, particularly reducing *Fto* expression while modulating other regulators toward SD levels. Quantitative RT-PCR analysis further validated these findings, showing significantly elevated *Fto* expression in HFD-fed mice, which was mitigated by treadmill exercise, while *Alkbh5* showed a non-significant increasing trend (Figure 4C). Additionally, exercise restored *Ythdf1* and *Ythdf2* expression, suggesting its role in reestablishing m6A-mediated RNA dynamics.

### 2.5. Treadmill Exercise Modifies the m6A RNA Methylation Landscape to Regulate Liver Function in HFD-Induced MASLD

**Figure 5:**
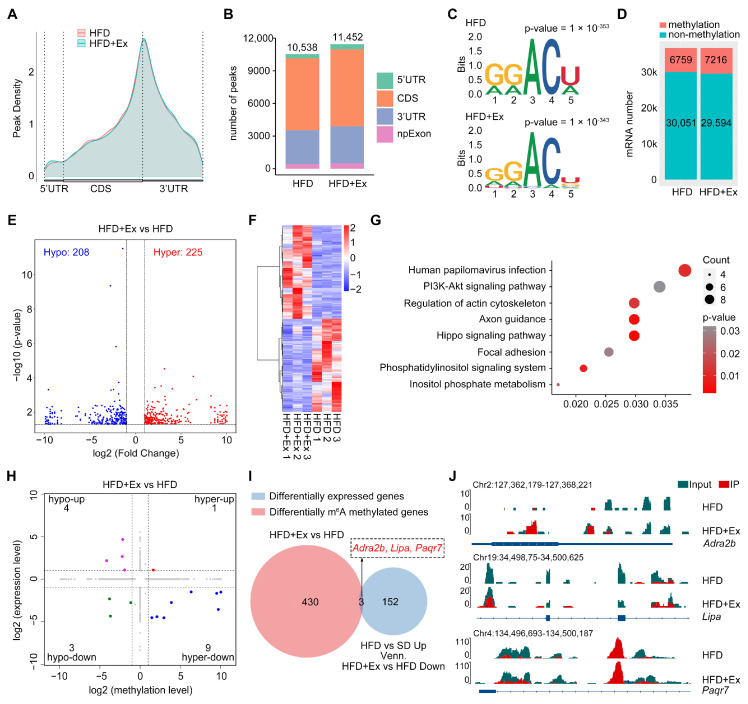
Treadmill exercise reshapes the m6A RNA methylation landscape in the liver of mice with HFD-induced MASLD. (**A**) Pea`k density analysis of m6A methylation across 5′ untranslated region (UTR), coding sequence (CDS), and 3′ UTR regions in the HFD and HFD + Ex groups, showing predominant localization in the 3′ UTR and CDS regions with minimal alterations between groups. (**B**) Total number of m6A peaks in liver samples from the HFD and HFD + Ex groups, revealing a slight increase in peaks in the 5′ UTR and CDS regions in the HFD + Ex group. (**C**) Sequence motif analysis of m6A peaks indicates a conserved RRACH (R = G, A; H = A, C, U) motif as the predominant m6A site in both HFD and HFD + Ex samples. (**D**) Distribution of methylated versus non-methylated mRNAs in the HFD and HFD + Ex groups. (**E**) Volcano plot highlighting differentially methylated genes (DMGs) between the HFD and HFD + Ex groups, with 225 hypermethylated and 208 hypomethylated genes. The horizontal dotted line represents the limit of statistical significance (corresponding to *p*-value = 0.05) and vertical dotted lines indicate log2 fold-change cutoff of 1 and −1. (**F**) Heatmap showing methylation patterns of DMGs in the HFD and HFD + Ex groups. (**G**) KEGG pathway enrichment analysis of DMGs, identifying key pathways such as PI3K-Akt signaling, Hippo signaling, and focal adhesion. (**H**) Scatter plot correlating differential gene expression and methylation levels between HFD and HFD + Ex groups. (**I**) Venn diagram showing the overlap of differentially expressed and methylated genes, with *Adra2b*, *Lipa*, and *Paqr7* as notable candidates. (**J**) Genomic tracks of m6A modifications in *Adra2b*, *Lipa*, and *Paqr7*, demonstrating exercise-induced alterations in methylation.

To investigate the potential role of m6A RNA methylation in mediating the beneficial effects of treadmill exercise on MASLD, we performed MeRIP-Seq to profile the m6A RNA methylation landscape. Peak density analysis revealed that m6A modifications were predominantly localized in the 3′ UTR and CDS regions, with treadmill exercise slightly increasing m6A peaks in the 5′ UTR and CDS (Figure 5A,B). The canonical RRACH sequence was identified as the conserved m6A motif across conditions (Figure 5C). A comprehensive comparison of methylated and non-methylated mRNA transcripts indicated an increase in methylated mRNAs in the HFD + Ex group compared to the HFD group (Figure 5D). Further analysis revealed that treadmill exercise resulted in significant changes in methylation status, with 225 genes hypermethylated and 208 hypomethylated in the HFD + Ex group compared to the HFD group (Figure 5E). Heatmap clustering of these differentially methylated genes (DMGs) demonstrated distinct methylation patterns between the HFD and HFD + Ex groups, indicating that treadmill exercise significantly reshaped the m6A methylation landscape (Figure 5F). Pathway enrichment analysis of these DMGs identified key metabolic and signaling pathways, including PI3K-Akt, Hippo signaling, and focal adhesion, which are critical in liver metabolism and MASLD progression (Figure 5G).

To investigate the interplay between m6A RNA methylation and gene expression, we analyzed the correlation between differential methylation and expression levels in the HFD + Ex versus HFD groups. Genes were classified into four categories based on their methylation and expression changes: hyper-up, hyper-down, hypo-up, and hypo-down. A small proportion of genes exhibited concordant changes in both methylation and expression, with one gene falling into the hyper-up category, nine genes in the hyper-down category, four genes in the hypo-up category, and three gene in the hypo-down category (Figure 5H). Importantly, among the 433 DMGs and 155 DEGs upregulated by HFD and downregulated by treadmill exercise, three overlapping genes, namely *Adra2b*, *Lipa*, and *Paqr7*, were identified (Figure 5I). Visualized methylation profiles of these genes confirmed exercise-induced regulatory changes (Figure 5J). Notably, *Adra2b*, *Lipa*, and *Paqr7* were all significantly downregulated in the HFD + Ex group, and MeRIP-seq analysis showed that each gene exhibited increased m6A methylation levels compared to the HFD group. Collectively, these findings demonstrate that treadmill exercise reshapes the m6A methylation landscape, modulates key metabolic pathways, and identifies potential therapeutic targets for MASLD.

### 2.6. Treadmill Exercise Modulates PAQR7 Expression and Lipid Metabolism in MASLD

**Figure 6:**
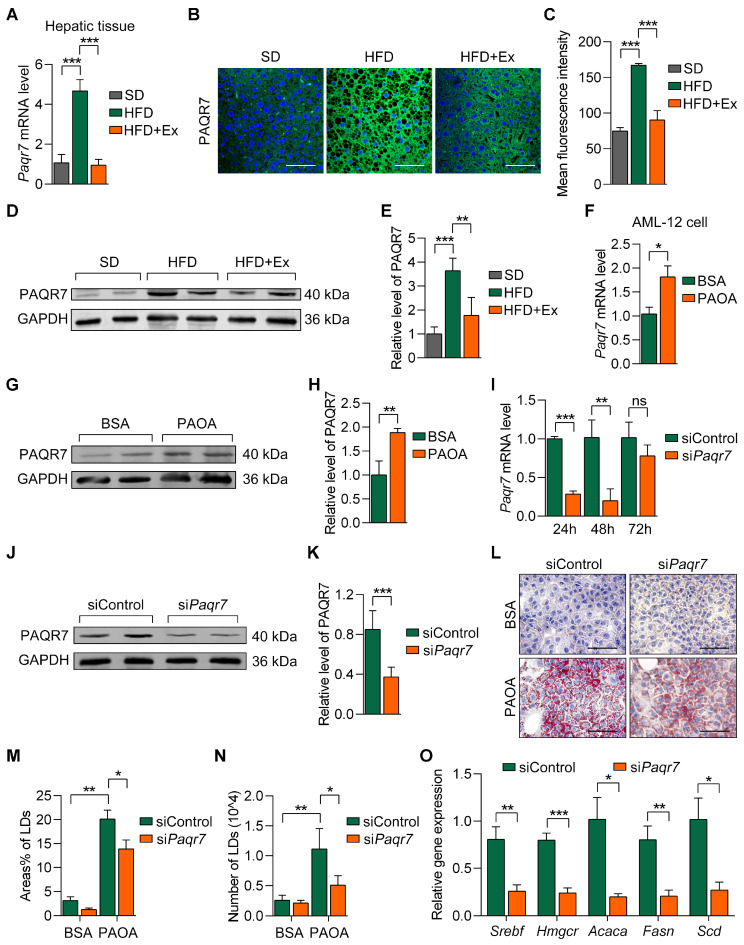
Treadmill exercise downregulates PAQR7 expression and modulates lipid metabolism in MASLD. (**A**) Relative mRNA expression of *Paqr7* in hepatic tissue from the SD, HFD, and HFD + Ex groups. (**B**) Representative immunofluorescence images showing PAQR7 expression in hepatic tissues across groups. Scale bars = 100 µm. (**C**) Quantification of mean fluorescence intensity of PAQR7 in (**B**). (**D**) Western blot and (**E**) quantification of PAQR7 protein levels in liver tissues from SD, HFD, and HFD + Ex groups. (**F**) Relative mRNA expression of *Paqr7* in AML-12 cells following palmitic acid and oleic acid (PAOA) treatment. (**G**) Western blot and (**H**) quantification of PAQR7 protein levels in AML-12 cells after PAOA treatments. (**I**) *Paqr7* mRNA expression in AML-12 cells following treatment with siPaqr7 at different time points (24 h, 48 h, 72 h). (**J**,**K**) PAQR7 knockdown validation by Western blot analysis in AML-12 cells after treatment with siPaqr7 for 24 h. (**L**) Oil Red O staining of AML-12 cells showing lipid droplet (LD) accumulation under siControl and siPaqr7 conditions with bovine serum albumin (BSA) or PAOA treatments. Scale bars = 100 µm. (**M**) Quantification of areas (% of LDs) in AML-12 cells from (**L**). (**N**) Quantification of the number of LDs per cell in (**L**). (**O**) Relative mRNA expression of lipogenic genes (*Srebf*, *Hmgcr*, *Acaca*, *Fasn*, *Scd*) in siControl and siPaqr7 groups under PAOA treatment. Data are presented as mean ± SD. * *p* < 0.05, ** *p* < 0.01, *** *p* < 0.001 by two-tailed *t*-test (**F**,**H**,**I**,**K**,**O**) or by one-way ANOVA (**A**,**C**,**E**,**M**,**N**).

The expression of *Paqr7* was markedly upregulated in the hepatic tissue of HFD-fed mice but was significantly reduced following treadmill exercise intervention, as evidenced by quantitative RT-PCR analysis (Figure 6A). Immunofluorescence staining further revealed elevated PAQR7 protein levels in the HFD group, which were notably reduced in the HFD + Ex group (Figure 6B,C). Western blot analysis confirmed these changes in PAQR7 protein expression across the groups (Figure 6D,E). In AML-12 cells, treatment with palmitic acid and oleic acid (PAOA) significantly increased *Paqr7* expression, as confirmed by both mRNA and protein level analyses (Figure 6F–H). Knockdown of *Paqr7* using siRNA (siPaqr7) effectively reduced *Paqr7* expression levels in AML-12 cells compared to the siControl group (Figure 6I–K). Subsequent Oil Red O staining revealed that PAQR7 knockdown significantly diminished LD accumulation and reduced LD area and number under PAOA treatment (Figure 6L–N). Moreover, *Paqr7* silencing downregulated key lipogenic genes, including *Srebf*, *Hmgcr*, *Acaca*, *Fasn*, and *Scd*, implicating its role in regulating lipid metabolism (Figure 6O). These results suggest that *Paqr7* plays a key role in lipid metabolism and that its regulation by exercise contributes to MASLD improvement.

## 3. Discussion

This study provides novel insights into the molecular mechanisms by which treadmill exercise may attenuate the progression of MASLD in an HFD-induced mouse model. Our findings suggest that exercise induces beneficial changes in liver function and metabolic parameters, which are associated with alterations in the hepatic m6A RNA methylation landscape. These changes highlight the potential role of RNA modifications in mediating the effects of environmental factors, such as exercise, on gene expression and liver pathology. The identification of m6A as a key regulatory mechanism linking exercise to gene expression related to lipid metabolism, oxidative stress, and inflammation represents a significant step forward in understanding the molecular underpinnings of exercise-induced liver protection.

The role of exercise in modulating liver function and metabolic health has been well documented, particularly in relation to improving insulin sensitivity, reducing fat accumulation, and ameliorating inflammation [21,22,23,24,25,26]. However, the molecular pathways through which exercise exerts these effects remain incompletely understood. Recent advances in epigenetics, particularly with respect to RNA modifications like m6A, have highlighted the complex ways in which gene expression can be regulated in response to environmental cues [9,20]. By showing that exercise alters hepatic m6A methylation, our study adds a new dimension to the existing body of literature, suggesting that exercise-induced epigenetic modifications may play a crucial role in the regulation of metabolic processes in the liver.

Previous study has shown that treadmill exercise can improve hepatic gene expression and metabolic outcomes in genetically modified models of MASLD [27]. However, our study adds to these findings by integrating transcriptomic and epitranscriptomic analyses to reveal RNA m6A methylation as a novel regulatory mechanism influenced by exercise. This perspective provides unique insight into how physical activity may mediate its beneficial effects on liver health at the RNA modification level. Our study provides a comprehensive examination of m6A methylation in the context of MASLD, with our findings representing the first detailed analysis of how exercise can reshape the m6A landscape in the liver. Using a combination of RNA-seq and MeRIP-Seq, we identified significant changes in m6A levels in genes involved in lipid metabolism, oxidative stress, and inflammatory pathways. These findings are consistent with previous research showing that m6A modifications regulate various aspects of cellular function, including RNA stability, translation, and splicing, all of which could contribute to the modulation of hepatic gene expression in response to exercise [28,29,30]. Specifically, the observation that exercise restores m6A levels in the liver to near baseline levels in HFD-fed mice suggests that m6A may function as a dynamic modulator of gene expression, responsive to environmental changes such as physical activity.

More importantly, we identified three candidate genes, *Adra2b*, *Lipa*, and *Paqr7*, that exhibited differential methylation and expression following exercise intervention, suggesting their potential roles in the prevention of MASLD. Among them, *Adra2b* encodes a G-protein-coupled receptor that plays a key role in catecholamine signaling, including the regulation of neurotransmitters such as norepinephrine and epinephrine [31]. While *Adra2b* may influence lipolysis and systemic metabolic processes, its expression in hepatocytes is relatively low, which likely limits its direct involvement in hepatic lipid metabolism. Thus, its effects are more systemic, impacting tissues like adipose tissue and skeletal muscle. Lysosomal Acid Lipase A (LIPA) is an enzyme primarily responsible for the hydrolysis of esterified cholesterol and triglycerides in the lysosome, playing a crucial role in lipid metabolism, and its protective role on HFD-induced liver damage has been well documented, particularly through studies using hepatocyte-specific knockout models [32,33]. *Paqr7*, a member of the progestin and adipoQ receptor family, is known for its role in mediating nongenomic progesterone actions, particularly in female reproductive tissue [34,35,36]. While *Paqr7* is expressed in hepatocytes, its role in MASLD has not been comprehensively elucidated. In our study, knockdown of *Paqr7* in AML-12 cells led to a significant reduction in lipid droplet accumulation and downregulated the expression of key lipogenic genes, suggesting that *Paqr7* may act as a critical mediator of hepatic lipid storage and metabolism. This positions *Paqr7* as a promising target for therapeutic intervention in MASLD. Future studies should aim to elucidate the precise role of *Paqr7* in MASLD using reliable genetic models, such as liver-specific knockouts or overexpression systems. Such investigations will be instrumental in confirming its mechanistic contribution to hepatic lipid metabolism and its potential as a therapeutic target in the context of exercise-mediated prevention of MASLD.

However, it is important to recognize that while our results provide compelling evidence for the role of m6A methylation in exercise-induced liver protection, this study has certain limitations. First, while we have demonstrated an association between exercise and changes in m6A methylation, this does not establish a direct causal relationship. Future studies that employ genetic or pharmacological manipulation of m6A “writers”, “erasers”, and “readers” will be necessary to confirm the specific role of m6A in mediating the beneficial effects of exercise on liver function. Such studies could help identify which genes are most critically regulated by m6A in the liver and whether these changes are directly responsible for the observed improvements in MASLD pathology.

Additionally, while our focus was on m6A, it is possible that other epigenetic mechanisms, such as DNA methylation, histone modifications, and non-coding RNA regulation, also contribute to the exercise-induced changes in liver gene expression. Recent studies have demonstrated that these modifications can interact with RNA methylation to modulate gene expression in a coordinated manner [37,38]. Therefore, future research should explore the interplay between m6A and other epigenetic marks to provide a more comprehensive understanding of how exercise impacts liver function. Furthermore, non-coding RNAs, particularly long non-coding RNAs and microRNAs, may also be involved in regulating m6A-modified transcripts, and their role in exercise-induced changes in liver metabolism warrants further investigation.

Another limitation of the current study is the use of a single exercise modality (treadmill running) and a specific animal model (C57BL/6 mice on a high-fat diet). While this model is widely used in MASLD research, it may not fully recapitulate the complexity of human MASLD, which is influenced by a wide range of factors, including genetic predisposition, diet, and comorbidities such as obesity and diabetes. In addition, we did not assess glucose metabolism through tests such as OGTT or ITT, which limits our understanding of systemic metabolic adaptations to exercise in this model. Furthermore, the lack of confirmatory genetic models, such as *AlbCrePten^flox/flox^* or *Fxr*^−/−^ mice, represents a further limitation, as these models may better capture the hereditary aspects of MASLD. Future studies should examine the effects of different exercise types (e.g., resistance training or high-intensity interval training) and consider using more diverse animal models to better represent the multifactorial nature of human metabolic diseases. In particular, human clinical studies are needed to determine whether the epigenetic modifications observed in the animal model are also relevant to human MASLD and to explore the potential for exercise-induced epigenetic changes to be used as biomarkers or therapeutic targets.

## 4. Materials and Methods

### 4.1. Animals Diet and Exercise Intervention

Six-week-old male C57BL/6 mice were housed under controlled conditions (23 ± 2 °C, 50% ± 5% humidity) with a 12-h light/dark cycle and ad libitum access to food and water. After a one-week acclimatization period, the mice were randomly assigned to one of three groups: standard diet control (SD, *n* = 6), sedentary high-fat diet (HFD, *n* = 6), and HFD with exercise (HFD + Ex, *n* = 6). The HFD and HFD+Ex groups were fed a high-fat diet (TP23400, Trophic Animal Feed High-Tech Co., Ltd., Nantong, China) containing 60% kcal from fat, 25.9% from carbohydrate, and 14.1% from protein. The standard diet used in the control group was LAD3001M from the same supplier, providing 10% kcal from fat. The exercise protocol involved 5 days of treadmill familiarization followed by 12 weeks of treadmill training. During the familiarization phase, running speed was gradually increased from 7 m/min to 13 m/min, with running duration extended from 15 to 60 min/day. During the formal training period, mice underwent 60-min sessions, comprising a 5-min warm-up (7 m/min), 60 min of running (13 m/min), and a 5-min cool-down (7 m/min), 5 days per week for 12 weeks. Body weight and food intake were measured weekly to monitor any potential confounding effects from the diet or exercise regimen. All animal experiments were approved by Fujian Normal University Institutional Animal Care and Use Committee (Approval No. IACUC-20240021).

### 4.2. Cell Culture and Treatment

Murine AML-12 hepatocytes cell line was obtained from the Cell Bank of the Type Culture Collection of the Chinese Academy of Sciences (Shanghai, China) and cultured in DMEM/F12 (1:1) medium supplemented with 10% fetal bovine serum (FBS), 5 µg/mL insulin, 5 µg/mL transferrin, 5 ng/mL selenium, 40 ng/mL dexametasona, and 1% penicillin–streptomycin. Cells were maintained at 37 °C in a humidified atmosphere of 5% CO_2_. For fatty acid stimulation, cells were treated with a mixture of oleic acid and palmitic acid (2:1 ratio) at a final concentration of 250 μM, complexed with 1% bovine serum albumin (BSA), for 24 h. siRNA transfection was performed as described below. Mouse *Paqr7* siRNA (5′-CCCGCTCTTCTCTATCACAAA-3′) and control siRNA were synthesized by Sangon Biotech (Shanghai, China). siRNAs were transfected into AML-12 cells at 60–80% confluence using LipofectamineTM 2000 Transfection Reagent (11668019, Invitrogen, Carlsbad, CA, USA) following the manufacturer’s protocol. Cells were harvested 24–72 h post-transfection for downstream analyses.

### 4.3. Physiological Measurements

Body weight was recorded weekly until the mice were sacrificed. For serum biochemical analysis, blood samples were centrifuged at 13,000 rpm for 15 min at 4 °C to obtain serum. Serum levels of alanine aminotransferase (ALT), aspartate aminotransferase (AST), triglycerides (TG), and total cholesterol (TC) were measured using an automatic biochemical analyzer (Chemray 800, Rayto, Shenzhen, China).

### 4.4. Histological Analysis

Liver tissues were immediately extracted, weighed to determine wet weight, and then imaged using an Axio Zoom.V16 Stereo Zoom Microscope with an Axiocam 506 Color camera (Carl Zeiss, Oberkochen, Germany). A consistent left lateral lobe of the liver from each animal was isolated, fixed in 4% paraformaldehyde (PFA) at 4 °C overnight, and embedded in paraffin. Sections (6 µm) were stained with hematoxylin and eosin (H&E) and Sirius Red. Another portion of the liver tissue was embedded in optimum cutting temperature (OCT) compound (Sakura Finetek, Tokyo, Japan) and sectioned for Oil Red O staining. Images were captured using a light microscope (Olympus BX51, Tokyo, Japan). NAFLD activity score (NAS) was evaluated as previously described [39], with steatosis (<5% = 0, 5–33% = 1, 33–66% = 2, >66% = 3), lobular inflammation (none = 0, <2 foci = 1, 2–4 foci = 2, >4 foci = 3), and hepatocellular ballooning (none = 0, few = 1, prominent = 2) being scored. NAS was calculated as the unweighted sum of these scores. Lipid droplet number and area were analyzed using Image Pro Plus 6.0 software (Media Cybernetics, Lehigh Valley, PA, USA). All scoring was performed in a blinded manner for each sample.

### 4.5. RNA Sequencing (RNA-Seq) Analysis

Total RNA was extracted from fresh mouse liver tissues using the RNeasy Mini Kit (74106, QIAGEN, Hilden, Germany) according to the manufacturer’s protocol. RNA concentration and purity were assessed using a NanoDrop 2000 spectrophotometer (Thermo Fisher Scientific, Waltham, MA, USA) and an Agilent 2100 Bioanalyzer (Agilent Technologies, Santa Clara, CA, USA). RNA sequencing was conducted on the Illumina NovaSeq platform. Sequenced reads quality was evaluated using FastQC (version 0.11.5), and raw reads were processed with Cutadapt (version 1.11) to remove low-quality reads and adapters. Clean reads were aligned to the mouse reference genome (mm10) using HISAT2 (version 2.0.1-beta) with default settings. Aligned reads were quantified using FeatureCounts (version 1.6.0), and expression levels were normalized and expressed as Reads Per Kilobase of transcript per Million mapped reads (RPKM). Differentially expressed genes (DEGs) between groups were identified using the edgeR package (version 3.12.1) with criteria of |log2FC| > 1 and adjusted *p*-value < 0.05. Functional annotation and pathway enrichment analyses of DEGs were performed using gene ontogeny (GO) and KEGG databases via DAVID Bioinformatics Resources (http://david.ncifcrf.gov (accessed on 24 December 2024)). Visualization of data was performed using the GOplot package in R.

### 4.6. m6A RNA Methylation Quantification

RNA m6A methylation levels were measured using the EpiQuik m6A RNA Methylation Quantification Kit (P-9005, Epigentek, Farmingdale, NY, USA) following the manufacturer’s protocol. Briefly, total RNA was extracted from liver tissues and immobilized onto strip wells using the provided RNA high-binding solution. Subsequently, a capture antibody solution and a detection antibody solution were sequentially added to the wells. The absorbance at 450 nm was measured using a spectrophotometer for colorimetric quantification. The m6A methylation content was determined based on the optical density (OD) values, which were directly proportional to the amount of m6A present.

### 4.7. Reverse Transcription and Quantitative PCR (RT-qPCR)

Total RNA was extracted from mouse liver tissue or AML-12 cells using the Qiagen RNeasy extraction kit, following the manufacturer’s protocol. The concentration, integrity, and purity of extracted RNA were assessed using a NanoDrop 2000 spectrophotometer (Thermo Fisher Scientific). Subsequently, 1 µg of total RNA was reverse-transcribed into complementary DNA (cDNA) using the SuperScript II Reverse Transcriptase Kit (18064014, Invitrogen). Quantitative real -time PCR (qRT-PCR) was performed using a fluorogenic SYBR Green master mix and the StepOnePlus Real-Time PCR System (Applied Bio-Systems, Waltham, MA, USA). Gene expression levels were normalized to the housekeeping gene GAPDH. Each reaction was run in triplicate and relative gene expression was calculated using the ΔΔCt method (2^−ΔΔCt^) [40]. Primer sequences are provided in Table S1.

### 4.8. Methylated RNA Immunoprecipitation Sequencing (MeRIP-Seq)

The MeRIP-Seq service was provided by SEQHEALTH Company (Wuhan, China). Total RNA was extracted from fresh mouse liver tissues using TRIzol reagent (9108, TaKaRa, Otsu, Japan) following the manufacturer’s protocol. Polyadenylated RNA was subsequently isolated using the Poly(A) RNA Selection Kit (NEB, MA, USA). The isolated RNA was fragmented into approximately 100-nucleotide fragments using RNA fragmentation buffer. For immunoprecipitation, fragmented RNA was incubated with an anti-m6A antibody (ABE572, Merck Millipore, Boston, MA, USA) in immunoprecipitation buffer and rotated at 4 °C for 2 h. The RNA-antibody complexes were captured using Protein A/G magnetic beads (Thermo Fisher Scientific) pre-blocked with BSA and yeast tRNA. Following extensive washing to remove non-specific bindings, m6A-enriched RNA was eluted and purified using a MinElute RNA Cleanup Kit (QIAGEN). Both m6A-enriched RNA and input RNA (as a control) were used to construct sequencing libraries with the NEBNext Ultra RNA Library Prep Kit (NEB, MA, USA). Libraries were quantified, pooled, and sequenced on an Illumina NovaSeq platform. Sequencing data were processed to align reads to the mouse reference genome (mm10) using HISAT2, and peak calling for m6A sites was performed with the exomePeak R package. Differential m6A peak analysis was conducted to identify condition-specific methylation changes.

### 4.9. Immunofluorescence

Paraffin-embedded liver tissue was sectioned into 6 µm for immunofluorescence analysis. The sections were deparaffinized in xylene and rehydrated through a graded ethanol series. Antigen retrieval was performed by incubating the sections in 10 mM sodium citrate buffer (pH 6.0) at 95 °C for 30 min. After antigen retrieval, the sections were blocked with 5% normal serum at room temperature for 45 min to reduce non-specific binding. Primary antibodies targeting PAQR7 (CPA7020, Cohesion Biosciences, London, UK) were applied overnight at 4 °C. Following primary antibody incubation, the sections were washed with PBST (PBS containing 0.1% Triton X-100) and incubated with Donkey anti-Rabbit IgG (H + L) Highly Cross-Adsorbed Secondary Antibody, Alexa Fluor™ 488 (A-21206, Invitrogen) for 1 h at room temperature. Nuclei were counterstained with DAPI (D1306, Thermo Fisher Scientific) for 10 min. After final washes with PBST, the sections were mounted with antifade mounting medium and imaged using a laser scanning confocal microscope (LSM780, Carl Zeiss).

### 4.10. Western Blot

Total protein was extracted from either liver tissues or AML-12 cells using RIPA buffer supplemented with 1% protease inhibitor cocktail (4693116001, Roche, Basel, Switzerland). The total protein concentration was quantified using a BCA Protein Assay Kit (23225, Invitrogen) according to the manufacturer’s protocol. Equal amounts of protein were separated by electrophoresis on 12% SDS-PAGE gels and transferred onto polyvinylidene fluoride (PVDF) membranes (ISEQ00010, Merck Millipore). The membranes were blocked with 5% BSA in TBS for 1 h at room temperature. Membranes were then incubated overnight at 4 °C with primary antibodies targeting PAQR7. After thorough washing with TBS containing 0.1% Tween-20, the membranes were incubated with fluorescently labeled secondary antibodies (LI-COR, Lincoln, NE, USA) for 1 h at room temperature. Protein bands were visualized using the Odyssey CLx imaging system (LI-COR), and band intensities were quantified using Image Studio Software (Version 3.1) (LI-COR). The GAPDH (AC033, ABclonal Technology, Wuhan, China) were used to normalize the amount of protein loaded.

### 4.11. Statistical Analysis

Statistical analysis was performed with GraphPad Prism 9.0 software. Replicate experiments were all biological replicates with different animals and data are presented as mean ± SD. Comparisons between two groups were performed using a two-tailed unpaired Student’s *t*-test. For multiple group comparisons, one-way analysis of variance (ANOVA) followed by Tukey’s post hoc test was applied when data met assumptions of normality and homogeneity of variance. When these assumptions were not met, the non-parametric Kruskal-Wallis test was used instead. A *p*-value of less than 0.05 was considered statistically significant.

## 5. Conclusions

In conclusion, our study provides new evidence that m6A RNA methylation plays a significant role in mediating the beneficial effects of exercise on liver function in MASLD. By identifying m6A as a key regulatory mechanism, we offer a deeper understanding of how exercise influences gene expression in the liver. These findings pave the way for future research into the therapeutic potential of modulating m6A methylation in the treatment of MASLD and other metabolic diseases. Further studies are needed to clarify the causal relationship between m6A modifications and the beneficial effects of exercise, as well as to explore the broader epigenetic landscape and its potential for therapeutic intervention.

## Data Availability

The original contributions presented in this study are included in the article/supplementary material. Further inquiries can be directed to the corresponding author.

## Supplementary Materials

The following supporting information can be downloaded at https://www.mdpi.com/article/10.3390/ijms26125810/s1. Table S1: The primers used in qRT-PCR analysis.

## Abbreviations

The following abbreviations are used in this manuscript:

ALT	Alanine aminotransferase
AST	Aspartate aminotransferase
DEG	Differentially expressed gene
DMG	Differentially methylated gene
HFD	High-fat diet
KEGG	Kyoto encyclopedia of genes and genomes
m6A	N6-methyladenine
MASLD	Metabolic-associated steatotic liver disease
NASH	Non-alcoholic steatohepatitis
NCC	Hepatocellular carcinoma
PAOA	Palmitic acid and oleic acid
SD	Standard diet
TC	Total cholesterol
TG	Triglycerides
