# Supplementary material for: Treadmill Exercise-Induced RNA m6A Methylation Modification in the Prevention of High-Fat Diet-Induced MASLD in Mice
Source: Int J Mol Sci. 2025 Jun 17;26(12):5810. doi: 10.3390/ijms26125810 (PMC12192837; doi:10.3390/ijms26125810)
Supplement: Supplementary file 1 [file ijms-26-05810-s001.zip › ijms-3680686-supplementary.pdf]

Supplementary data

Table S1. The primers used in qRT-PCR analysis

Gene	Forward primer	Reverse prime
Mettl3	CTGGGCACTTGGATTTAAGGAA	TGAGAGGTGGTGTAGCAACTT
Mettl14	CTGAGAGTGCGGATAGCATTG	GAGCAGATGTATCATAGGAAGCC
Wtap	GAACCTCTTCCTAAAAAGGTCCG	TTAACTCATCCCGTGCCATAAC
Fto	TCCTCAGAAGATGCCCTACTTG	CCCAACATTACCCAGCATGAAA
Alkbh5	CGCGGTCATCAACGACTACC	ATGGGCTTGAACTGGAACTTG
Ythdf1	ACAGTTACCCCTCGATGAGTG	GGTAGTGAGATACGGGATGGGA
Ythdf2	GAGCAGAGACCAAAAGGTCAAG	CTGTGGGCTCAAGTAAGGTTC
Ythdf3	CATAGGGCAACAGAGGAAACAG	ATCTCCAGCCGTGGACCAT
Ythdc1	GTCCACATTGCCTGTAAATGAGA	GGAAGCACCCAGTGTATAGGA
Ythdc2	ACCGACTAAGTCAATCTCTTGGT	AGGCTCCTAACAGCATGTTTTG
Srebf	TGGAGCTTTTGAGACTCAGGA	TCGATTAAGCAGGTGAGGTCG
Hmgcr	GCTTGGCCTCCATTGAGAT	ATGCATCCGGAAAAGTCTTG
Acaca	GATGAACCATCTCCGTTGGC	GACCCAATTATGAATCGGGAGTG
Fasn	GGCTCTATGGATTACCCAAGC	CCAGTGTTCGTTCCTCGGA
Scd	TTCTTGCGATACACTCTGGTGC	CGGGATTGAATGTTCTTGTCGT
Paqr7	TCTTCTTGGACTATGTGGGTGT	CTGCACCTTGTCATGCCAG
